# Development of the low back health awareness in patient transfer scale for operating room and intensive care unit staff and evaluation of factors associated with low back pain

**DOI:** 10.12669/pjms.42.4.14381

**Published:** 2026-04

**Authors:** Kamber Kasali, Habip Burak Ozgodek, Aysenur Dostbil, Sabri Selcuk Atamanalp

**Affiliations:** 1 Kamber Kasali, MD Assistant Professor, Department of Biostatistics, Faculty of Medicine, Ataturk University, Erzurum, Turkiye; 2 Habip Burak Ozgodek, MD Clinic of Anaesthesiology and Reanimation, Erzurum City Hospital, Erzurum, Turkiye; 3 Aysenur Dostbil, MD Professor, Department of Anaesthesiology and Reanimation, Faculty of Medicine, Ataturk University, Erzurum, Turkiye; 4 Sabri Selcuk Atamanalp, MD Professor, Department of General Surgery, Faculty of Medicine, Ataturk University, Erzurum, Turkiye

**Keywords:** Low back pain, Health awareness, Scale development, Patient transfer, Healthcare workers

## Abstract

**Objectives:**

Low back pain is a widespread musculoskeletal problem that causes significant social and economic burdens by leading to loss of working capacity and functional impairment. Those who transfer patients, particularly operating theatre and intensive care staff, are at high risk. This study aimed to develop the low back health awareness in patient transfer scale (LBHAPTS) and evaluate the prevalence, risk factors and consequences of back pain among these personnel.

**Methodology:**

This methodological study developed the LBHAPTS through item generation, content validity, pilot testing, factor analyses (EFA, CFA), and reliability testing, with data from 288 healthcare workers.

**Results:**

The 22-item scale demonstrated high validity (S-CVI/Ave = 95.45%) and reliability (Cronbach’s α = 0.910; ICC = 0.882). Factor analyses confirmed a three-factor structure explaining 57% of the total variance. Model fit indices were acceptable (χ^2^/df = 2.45, CFI = 0.907, RMSEA = 0.071). Scale scores showed significant differences according to education, equipment, and back health knowledge, with no differences found between occupational groups.

**Conclusion:**

Our study concludes that the scale is a useful and reliable tool, highlighting the importance of training and knowledge to reduce ergonomic risks and promote safe patient transfer practices.

## INTRODUCTION

Low back pain (LBP) is a prevalent musculoskeletal condition worldwide, imposing substantial social and economic burdens through reduced productivity and functional impairment.^1^,^2^ Healthcare workers, particularly those working in operating theatres and intensive care units, are at higher risk due to frequent patient transfers and repetitive movements in awkward positions.^3^-^5^ Limited access to mechanical lifting devices often necessitates manual lifting, increasing the incidence of LBP.^6^ Prevalence among nurses ranges from 40% to 97.9%, exceeding 80% in intensive care nurses.^1^,^6^,^7^ Risk factors include age, female sex, obesity, smoking, sedentary lifestyle, prolonged standing, heavy lifting, and repetitive bending twisting, as well as workplace issues like high stress, heavy workload, and inadequate staffing.^3^,^6^

These conditions are common in operating room and intensive care settings. Occupational LBP reduces productivity, increases absenteeism, and can lead to early retirement, causing loss of experienced staff, heavier workloads, and diminished care quality.^3^,^8^,^9^ Treatment and rehabilitation costs also burden individuals and institutions.^6^,^10^ This study primarily aimed to develop the low back health awareness in patient transfer scale (LBHAPTS) to assess healthcare personnel’s awareness of spinal health protection during transfers. Secondarily, it evaluates the prevalence, risk factors, and outcomes of LBP among resident physicians, anaesthesia technicians, nurses, and auxiliary staff involved in transfers, contributing to preventive strategies and safe patient handling practices.

## METHODOLOGY

This study was performed on healthcare staff working in the operation rooms of Ataturk University Faculty of Medicine Research Hospital. This methodological study used a descriptive survey design to develop the LBHAPTS, following the steps of Polit and Beck^11^ in 2006 and DeVellis and Thorpe^12^ in 2022. Our sample comprised 288 healthcare workers.

### Ethical Approval:

Prior to the commencement of the study, ethical approval was obtained from the Ethics Committee of Atatürk University (Ethics Committee Decision No: B.30.2.ATA.0.01.00/130, Date: 28 February 2025). Each participant signed an informed consent form attesting to their voluntary involvement.

### Scale Development Stages:

### Item Generation:

Based on a literature review and expert opinions from academic and clinical professionals, a total of 25 items were developed. The items were designed using a 5-point Likert (1 = Strongly Disagree, 5 = Strongly Agree).

### Content Validity:

As a result of content validity assessed by five experts, three items were removed, and the I-CVI values of the remaining 22 items were found to be between 80% and 100%. 77.3% of the items (I-CVI = 100%) were approved by all experts, the scale-level S-CVI/Ave was calculated as 95.45%, exceeding the recommended threshold of 90% and demonstrating high content validity.^11^

### Pre-testing and Pilot Study:

The draft 22 item scale was administered to a small group of 10 healthcare workers to assess clarity and applicability, after which necessary linguistic and wording adjustments were made. These individuals were not included in the main study sample.

### Item and Factor Analysis:

Exploratory factor analysis indicated that the data were suitable for factorisation (KMO = 0.954; Bartlett p < 0.001). Three factors with eigenvalues above 1 explained 57.1% of the total variance. Factor loadings ranged from 0.531 to 0.829. Confirmatory factor analysis showed good fit (χ^2^/df = 2.45; CFI = 0.907; TLI = 0.892; RMSEA = 0.071). All factor loadings were shown to be significant (p < 0.001).

### Reliability Analysis:

The overall Cronbach’s alpha value for the scale was 0.912. The alpha values for the sub-dimension were 0.897, 0.854, and 0.818, respectively. Split-half reliability analysis yielded a Spearman-Brown coefficient of 0.706 and a Guttman Split-Half coefficient of 0.706. The Intraclass Correlation Coefficient (ICC) was calculated as 0.882 (95% CI: 0.854-0.905, p < 0.001).

### Validity Analysis:

Construct validity was confirmed through both EFA and CFA. The inter-item relationships and factor structure demonstrated that the scale appropriately measures the intended construct.

### Sample Size and Statistical Analysis:

The sample size was calculated using Cronbach’s α. With 95% confidence and 80% power, assuming a null α of 0.80 and a target α of 0.845, at least 250 participants were required, calculated using formulas from the Real Statistics website. Ultimately, 288 healthcare workers participated voluntarily.

Data were analysed using SPSS 20.0 and JAMOVI 2.2.2. Categorical variables were presented as frequencies and percentages; continuous data as mean ± SD; Median. Normality was checked using Shapiro-Wilk. Between-group comparisons employed the t-test or Mann-Whitney U, and for more than two groups, ANOVA or Kruskal-Wallis with appropriate post hoc tests. Validity and reliability were assessed through expert review, KMO, Bartlett’s test, scree plot, and both Exploratory and Confirmatory Factor Analyses with fit indices (χ^2^/df, RMSEA, SRMR, TLI, CFI). Reliability was further confirmed using Cronbach’s α, split-half, Guttman coefficient, and Hotelling’s T². Multivariate normality in CFA was verified by Mardia’s test, and significance was set at p < 0.05.

## RESULTS

### Descriptive Statistics of the Variables:

Total 288 individuals participated in this study. Mean age was 33 ± 8 years. Mean professional experience period and mean working year in healthcare were 10 ± 7 years. Average patient transfer count was 5 ± 5.

As presented in Table-I, 150 (52.1%) of the individuals were male and 138 (47.9%) were female. 17.4% of participants were high school graduates, 31.9% were associate degree holders, 28.5% were bachelor’s degree holders, and 22.2% were master’s degree holders. Most (83.3%) had patient transfer experience, and 44.8% had a history of back problems. Only 22.6% of participants had received relevant training, while 68.1% knew about transfer equipment and 40.3% knew about back health protection methods. The occupational groups were distributed as follows: assistant physicians (17.7%), nurses (27.8%), anaesthesia technicians (32.3%), and support staff (22.2%).

**Table-I:** Descriptive statistics of the variables.

Parameter		n
Gender	Male	150 (52.1%)
	Female	138 (47.9%)
Educational status	High school	50 (17.4%)
	Associate degree	92 (31.9%)
	Licence	82 (28.5%)
	Master’s degree	64 (22.2%)
Patient transfer experience	Yes	240 (83.3%)
	No	48 (16.7%)
History of low back pain	Yes	129 (44.8%)
	No	159 (55.2%)
Training received	Yes	65 (22.6%)
	No	223 (77.4%)
Knowledge of equipment used in transfer	Yes	196 (68.1%)
	No	92 (31.9%)
Knowledge of low back health protection	Yes	116 (40.3%)
	No	172 (59.7%)
Healthcare profession	Assistant doctor	51 (17.7%)
	Nurse	80 (27.8%)
	Anesthesia technician	93 (32.3%)
	Staff member	64 (22.2%)

### Content Validity Indices (I-CVI and S-CVI) Results of Scale:

Content validity was evaluated by Item-Level (I-CVI) and Scale-Level (S-CVI) indices based on expert evaluations. I-CVI values ranged from 80% to 100%, with 77.3% of items scoring 100%. The overall S-CVI/Ave was calculated as 95.45%, exceeding the 90% reference value and confirming high content validity.^9^ (Table-II).

**Table-II:** Content validity indices (I-CVI and S-CVI) results of scale.

Item	Detail	Number of experts giving a score of 3 or 4 (5 experts)	I-CVI
1	I am aware that incorrect techniques during patient transfers can threaten my back health.	5	100%
2	Before the transfer, I make sure that the operating table and stretcher are aligned.	4	80%
3	Before transporting the patient, I plan with my team.	5	100%
4	During the transfer, I move in a controlled manner, avoiding quick and sudden movements.	5	100%
5	If no suitable assistive device is available, I seek alternative safe solutions for patient	4	80%
6	During patient transfer, I move in an organised manner with my team.	5	100%
7	During transfer, we designate a leader in advance as a team.	5	100%
8	We communicate as a team before moving the patient during transfer.	5	100%
9	I try never to act alone during patient transfers.	5	100%
10	We count together as a team during patient transfer and move at the same time.	4	80%
11	I use assistive devices such as sliding boards or air-supported mats during patient transfer	5	100%
12	The institution I work for encourages the use of assistive devices during patient transfer	5	100%
13	I share the importance of patient lifting devices with my team.	5	100%
14	I do regular exercise to protect my back health.	5	100%
15	I increase my flexibility by doing stretching exercises before and after work.	4	80%
16	When I experience back pain, I take the necessary precautions and seek medical assistance.	5	100%
17	My workplace organises training courses on ergonomics and patient transfer.	5	100%
18	I make sure to attend training courses to learn the correct techniques.	5	100%
19	I take care to protect my back during patient transfers.	5	100%
20	I know and apply methods that reduce risks during patient transfers.	4	80%
21	When transferring patients, I lift by bending my knees, not my back.	5	100%
22	During patient transfers, I align my body correctly to avoid lifting uneven loads.	5	100%
S-CVI			95.45%

### Reliability Statistics of Scale:

The overall Cronbach’s alpha was 0.910, with subscale alphas of 0.897, 0.854, and 0.818, indicating high internal consistency. Split-half analysis yielded alphas of 0.885 and 0.869, an inter-form correlation of 0.546, and Spearman-Brown and Guttman coefficients of 0.706. Hotelling’s T² was significant (p < 0.001), and the ICC was 0.882 (95% CI: 0.854-0.905, p < 0.001), confirming strong reliability and stability (Table-III).

**Table-III:** Reliability statistics of scale.

Reliability statistics		Value	n
Cronbach’s Alpha (total)		0.910	22
Factor 1		0.897	10
Factor 2		0.854	8
Factor 3		0.818	4
Cronbach’s Alpha	Part 1	0.885	11
	Part 2	0.869	11
Correlation between forms		0.546	22
Spearman-Brown coefficient	Equal length	0.706	22
	Unequal length	0.706	22
Guttman Split-Half coefficient		0.706	22
Hotelling’s T-Squared test		Test value	569.573
		F	25.232
		p	<0.001
Intraclass correlation coefficient		Test value	0.882 (0.854-0.905)
		p	<0.001

### Exploratory Factor Analysis of Scale:

**Fig.1:**
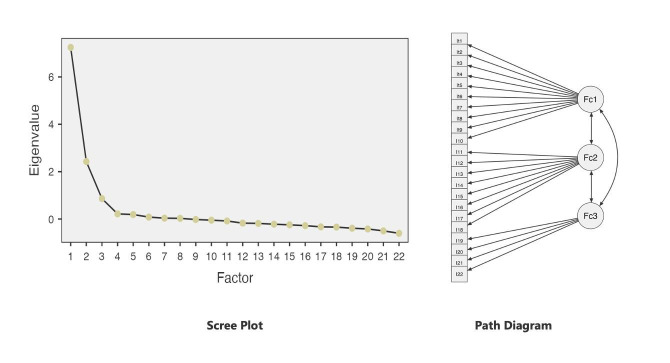
Scree plot and path diagram.

The communalities of the items ranged from 0.390 to 0.718, indicating that each item was moderately to highly represented by its respective factor. The lowest communality value was found for Item 11, whereas the highest was for Item six. After Varimax rotation, factor loadings varied from 0.531 to 0.829, and all items exceeded the minimum acceptable loading of 0.30. The analysis revealed a three-factor structure explaining 57.053% of the total variance. Based on expert opinions, Factor 1 (Items 1-10) was named “Team-Based Safe Transfer Practices,” Factor 2 (Items 11-18) “Institutional Support and Protective Practices,” and Factor 3 (Items 19-22) “Individual Transfer Skills and Body Mechanics”. The scree plot showed a noticeable levelling-off beginning with the third component, confirming the three-factor solution (Fig.1).

### Confirmatory Factor Analysis of Scale:

Confirmatory factor analysis shown acceptable model fit (χ^2^/df = 2.45, p < 0.001). While the χ^2^/df ratio indicated good fit, RMSEA (0.071; 90% CI: 0.063-0.079) and SRMR (0.074) were within acceptable ranges. CFI (0.907) and TLI (0.892) also met acceptable thresholds, confirming that the model fit the data well (Table-IV, Fig.1).

**Table-IV:** The goodness of fit results of scale and reference values.

Indexes reference value	Good fit	Acceptable fit	Measurement	Result
CMIN/DF	0 < χ 2/SD ≤ 3	3 < χ 2/SD ≤ 5	2.45	Good fit
RMSEA (90% CI)	0 ≤ RMSEA ≤.05	0.05 < RMSEA ≤.08	0.071 (0.063- 0.079)	Acceptable fit
SRMR	0 ≤ SRMR ≤.05	0.05 < SRMR ≤.10	0.074	Acceptable fit
CFI	0.95 < CFI ≤ 1	0.90 < CFI ≤.94	0.907	Acceptable fit
TLI	0.95 < TLI ≤ 1	0.90 < TLI ≤.94	0.892	Low fit
χ^2^	489			
df	199			
p	<0.001			

### Comparison of Scale Subdimensions and Total Score by Variables:

Significant differences were found in Team-Based Safe Transfer Practices scores based on patient transfer experience (p = 0.007), equipment knowledge (p = 0.007), back health protection knowledge (p < 0.001), and education level (p = 0.010). In Institutional Support and Protective Practices, receiving training (p = 0.001) and knowledge of back health protection (p < 0.001) were significant. In Individual Transfer Skills and Body Mechanics, training (p < 0.001), equipment knowledge (p = 0.006), and back health protection knowledge (p < 0.001) made a difference. In the total score, training (p < 0.001), equipment knowledge (p = 0.006), knowledge of back health protection (p < 0.001) and education level (p = 0.013) were found to be significant.

No statistical differences were detected between occupational groups in Team Based Safe Transfer Practices (p = 0.121), Institutional Support and Protective Practices (p = 0.122), Individual Transfer Skills and Body Mechanics (p = 0.115), or total score (p = 0.063) (p < 0.05). Table-V.

**Table-V:** Comparison of healthcare profession with the total scale score and subdimensions.

Parameter	Assistant Doctor	Nurse	Anesthesia Technician	Staff Member	p
	Mean ± SD ; Median	Mean ± SD ; Median	Mean ± SD ; Median	Mean ± SD ; Median	
Team based safe transfer practices	36.08 ± 8.44 ; 38	38.19 ± 6.09 ; 39	39.40 ± 7.70 ; 40	37.87 ± 9.31 ; 39.5	0.121^∂^
Institutional support and protective practices	20.14 ± 6.95 ; 19	22.00 ± 6.60 ; 21	21.33 ± 6.86 ; 22	23.02 ± 7.44 ; 23	0.122^∂^
Individual transfer skills and body mechanics	12.29 ± 4.03 ; 12	13.65 ± 3.13 ; 14	13.78 ± 3.64 ; 14	13.28 ± 3.84 ; 13	0.115^∂^
Total score	68.51 ± 14.15 ; 68	73.84 ± 13.04 ; 73	74.52 ± 14.90 ; 78	74.17 ± 17.30 ; 76	0.063^∂^

∂: Kruskal Wallis test.

## DISCUSSION

This study validated the LBHAPTS, designed to assess spinal health awareness among healthcare workers in operating rooms and intensive care units. The scale showed high internal consistency (Cronbach’s α = 0.91) and a three-factor structure confirmed by exploratory and confirmatory factor analyses.^13^ In our sample, 44.8% reported a history of low back problems, aligning with Kabeer et al,^14^ who found a 40-60% prevalence among healthcare workers. Consistent with previous studies linking patient transfer frequency to low back pain via ergonomic risks such as heavy lifting, poor posture, and lack of equipment use, 31.9% of participants reported not using transfer equipment.^15^-^17^ Only 22.6% had received relevant training, despite evidence that education and awareness programs reduce spinal health problems.^13^,^14^ In this study, training, equipment knowledge, and especially knowledge of low back health protection significantly improved subscale and total scores, indicating better safe transfer performance.

### Limitations:

This cross-sectional study reflects the situation at a single point in time, preventing causal inferences. As the sample was limited to personnel in Erzurum, generalizability to other regions is restricted. Self-reported data may involve social desirability or recall bias. The relationship between awareness and pain severity was not assessed with objective measures, and psychosocial factors or variations in working conditions were not analysed.

## CONCLUSIONS

This study demonstrates that the LBHAPTS is a valid and reliable tool for measuring healthcare workers’ awareness of spinal health. Furthermore, the findings indicate that training, equipment knowledge, and especially knowledge of low back health protection reduce risks during patient transfers. Overall, the results emphasize the need to minimize ergonomic risks and expand educational programs to promote safe patient handling practices.

### Author’s Contribution:

**KK:** Project development, data collection, manuscript writing, manuscript editing, and data analysis.

**HBO:** Data collection, revision of the final draft.

**AD:** Project development, data collection, revision of the final draft.

**SSA:** Project development and Manuscript writing. (scientific), AD (ethical), and KK (statistical) are responsible and accountable for the accuracy and integrity of the work.
